# Open hardware: From DIY trend to global transformation in access to laboratory equipment

**DOI:** 10.1371/journal.pbio.3001931

**Published:** 2023-01-17

**Authors:** Tobias Wenzel

**Affiliations:** Institute for Biological and Medical Engineering, Schools of Engineering, Medicine and Biological Sciences, Pontificia Universidad Católica de Chile, Macul, Región Metropolitana, Chile

## Abstract

Open hardware solutions are increasingly being chosen by researchers as a strategy to improve access to technology for cutting-edge biology research. The use of DIY technology is already widespread, particularly in countries with limited access to science funding, and is catalyzing the development of open-source technologies. Beyond financial accessibility, open hardware can be transformational for the access of laboratories to equipment by reducing dependence on import logistics and enabling direct knowledge transfer. Central drivers to the adoption of appropriate open-source technologies in biology laboratories around the world are open sharing, digital fabrication, local production, the use of standard parts, and detailed documentation. This Essay examines the global spread of open hardware and discusses which kinds of open-source technologies are the most beneficial in scientific environments with economic and infrastructural constraints.

## Abstract 2

Open hardware solutions are increasingly being chosen by researchers as a strategy to improve access to technology for cutting-edge biology research. This Essay discusses the current global reach and impact of open hardware, as well as suggesting solutions suitable for biology labs.

## Introduction

Most scientists who conduct experiments in research laboratories face at least some resource constrains such as access to specific know-how, technologies, equipment, and reagents. Given the vast complexity of biological systems and the demands of scientific rigor and speed, these technological constraints can quickly become a research-limiting challenge for academic laboratories engaged in knowledge generation and publication in the life sciences. If even well-funded research hubs struggle with access to technology, how do research laboratories in scientific environments characterized by economic and infrastructural constraints, here referred to as “low-resource settings,” deal with these challenges and still produce globally relevant research outputs? Do low-cost DIY approaches and free and open-source hardware (here “open hardware” or “open technology”) improve a researcher’s access to technology?

The aim of this Essay is to give an account of how DIY and open technologies are being implemented and taken up by researchers globally, and what effect this has on researchers with restricted access to technologies. These low-resource setting constraints extend beyond monetary considerations into a less-rich research and technology ecosystem, and, therefore, solutions are not only driven by cost but also by other factors such as local fabrication, access to parts, and direct knowledge transfer. Much of the reasons driving technology adoption in low-resource settings also apply to laboratories in general, where the unique advantages of open technology are used to address new custom research questions, to apply existing methods in new settings, and to increase the accessibility of methods.

### How does DIY hardware affect access to technology?

The idea of building your own equipment is as old as experimental science. However, in the context of the current, professionalized, commercial research laboratory infrastructure, the concept of researchers building their own laboratory tools is only beginning to be rediscovered. As of 21 November 2022, the Web of Science indexed 58,889 publications on 3D printing (77% since 2018) across all search fields; 19,308 on open hardware (47% since 2018); 10,196 mentioning Arduino microcontrollers (58% since 2018); and 6,010 mentioning the low-cost Raspberry Pi computer (70% since 2018). These terms encompass popular methods or tools for DIY instrumentation (Box 1) that are seeing a rapid growth in publication numbers. The portfolio of low-cost tools available is mostly driven by the wider “maker” movement outside of academia, where people are investing in their local production skills to create and customize technologies. An important aspect of the maker trend is the access to digital fabrication, which allows the interchange of increasingly complex digital designs that can be produced locally with an automated fabrication machine. Digital fabrication includes 3D printing, laser cutting, CNC milling, plotter cutting, the fabrication of electric circuit boards, and even microfluidic chips and DNA synthesis. Communal workshops such as FabLabs, MakerSpaces, and others have sprung up around the globe to provide access to digital fabrication.

**Box 1. Glossary**

**Arduino microcontrollers**

Microcontrollers are small circuit boards that contain processing core(s), memory, and input/output ports. Microcontrollers can be programmed by uploading code onto the board and are therefore used as “brains” in the electrical interiors of instrumentation. The Arduino project is not only open source; it also makes the use of its microcontrollers very easy, which explains its popularity, with more than 10 million of their Arduino Uno boards sold by 2021 [1]. Arduinos come in a standard form factor, do not need a separate programming device apart from a USB cable, and come with a software interface that allows the use of a simplified C++ programming language.

**CNC milling**

CNC (computer numerical control) means automation of machining tools such as drills, printers, routers, lathes, mills, and cutters, but it is usually only used in practice as part of the machine name when it comes to mills (for metal and tough materials), routers (for wood and softer materials), and lathes (for cylindrical objects and rotation). CNC mills, routers, and lathes are the key digital fabrication tools for materials that cannot be easily processed on low-cost 3D printers or laser cutters. They are common in most countries and, therefore, suitable to local fabrication through local services.

**Laser cutting**

Laser cutters are automated machines that can cut and engrave small and large sheets of acrylic, wood, or paper according to a 2D digital design. These digital fabrication machines are very fast, easy to use, and part of the basic inventory of most FabLabs and Maker Spaces. Cut 2D sheets of acrylic can be connected to assemble 3D objects, which is particularly practical for medium and large enclosures that would take a long time to 3D print, if they fit the printer at all.

**Raspberry Pi computer**

Raspberry Pis are popular low-cost single board computers with a small form factor that can run a Linux operating system (>37 million Raspberry Pis sold by 2020; [2]). Though not open source themselves, they are often used as computers inside open source instruments. There is a large community of users and a wealth of educational material available. They are ideal to run Python software to control equipment and provide a user interface.

**3D printing**

The open source RepRap project can be credited with the early development of low-cost open source 3D printers that enabled the current generation of models that are available in many homes, workshops, and labs around the world. While there is also an industrial branch of 3D printing, in the context of open laboratory instruments, we usually refer to the most common low-cost desktop printers: filament deposition modeling (FDM) printers that use cheap plastic filament spools (usually PLA material) to recreate 3D objects from digital files inside the 3D printer, layer-by-layer.

**Fig 1:**
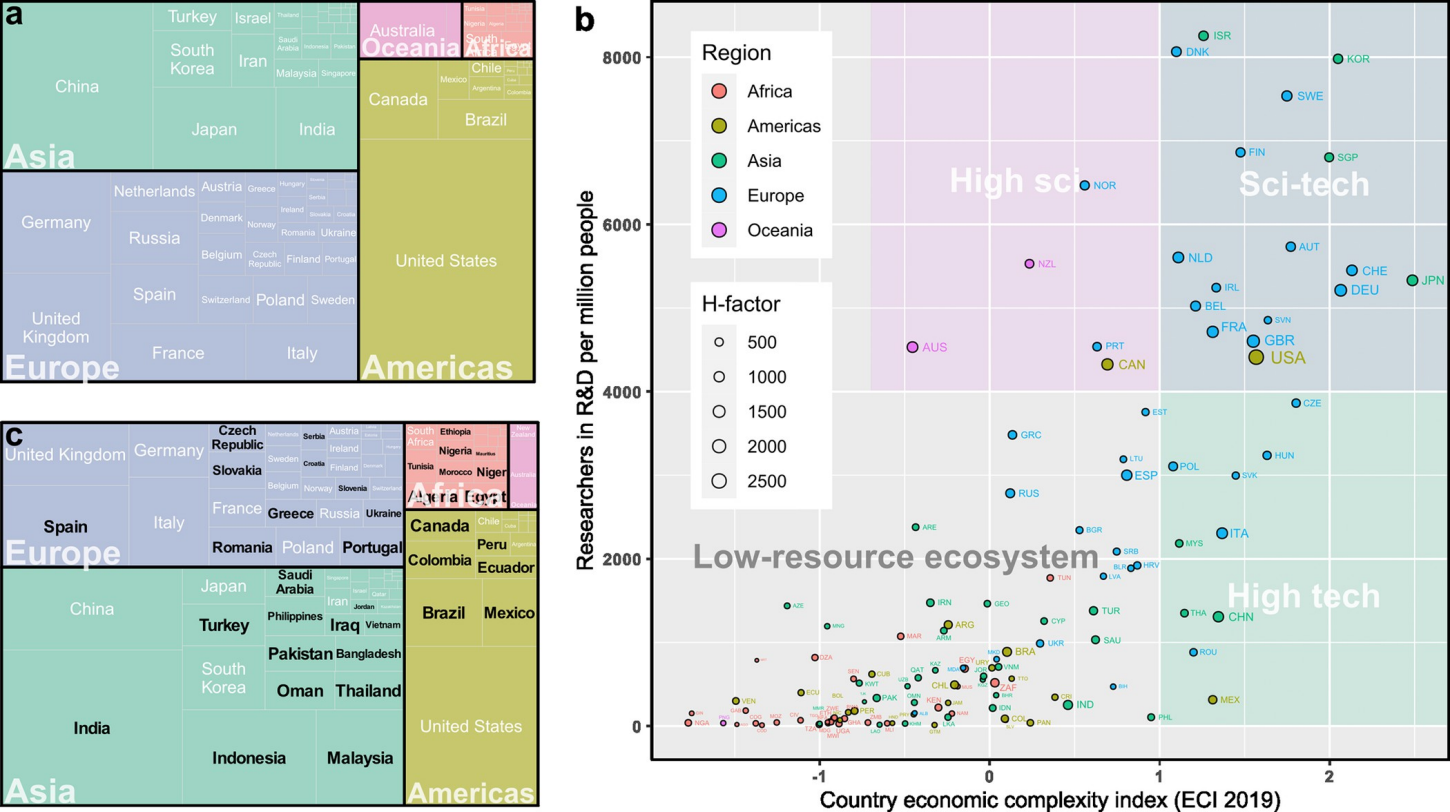
Publication statistics reveal global trends in DIY technology use. (**a)** The global distribution of total publications produced by countries and regions. A country’s fraction of total publications (58,485,740) is represented by its relative size in the graph. Data source: scimagojr total citable documents on any topic from 1996 to 2020. (**b)** Graph showing each country (marked by country code and colored by region) with respect to their density of researchers in the population and their economic complexity index. The size of the points represents the country’s H-index. Data sources: researcher density, Worldbank Development Indicators, latest data available by country; economic complexity index, Atlas of Economic Complexity, 2019 data or latest available; country H-index, scimagojr country rank, 1996–2020 all subjects; global regions, UN sustainable development goals. Countries without data in all relevant datasets were removed. (**c)** Global distribution of publications that mention the tools “Raspberry Pi” or “Arduino” in the method section of the article by country and regions. A country’s fraction of total publications (3,718) is represented by its relative size in the graph. Countries visibly overrepresented in this category relative to overall publications are highlighted in black. Data source: Web of Science (WoS) search results on “Raspberry Pi” or “Arduino” across publications (articles, proceedings papers, early access, and data papers) indexed before the search date (15 September 2022). A total of 13,285 articles were found. This publication dataset was then compared to a Scite search on wider literature with “Arduino” or “Raspberry Pi” in the method section (23,703 results). The datasets were then merged by DOI and, where absent, by title to obtain 3,718 matches that contain the WoS publication type filter, method section criterion, and publication country data. The relative publication by country distribution for all WoS articles on the topic is similar to the result displayed for the method section only. The R code to generate Fig 1 is shared in the following repository: https://osf.io/59bv2/.

The impact of DIY technologies on research can be assessed by analyzing recent scientific publications. When it comes to scientific publications in general, a few countries dominate the global ranking for number of manuscripts authored, as illustrated in Fig 1A. These countries typically have a high density of scientists and an industrial economy that produces complex goods, as can be seen in Fig 1B. But what about publications mentioning DIY tools? Whereas 3D printing or open hardware may be referred to in an industrial or theoretical context, the Arduino and Raspberry Pi boards are specific tools that are more likely to have been used in practice when mentioned as part of a study’s methodology. In this sense, publications using these terms can be used as a proxy for studies using DIY hardware in general. When searching for Arduino or Raspberry Pi keywords in the methods section of articles (Fig 1C), the publication distribution reveals interesting differences in comparison to the overall publications (Fig 1A). The countries visibly overrepresented among those publishing studies that mention DIY technology tend to be located in regions of the world with fewer science resources. With little comparative data available on laboratory technology use, this data provides an indicator that DIY technology does indeed contribute to technology access in low-resource settings. Unfortunately, many studies in biological sciences that use such tools do not highlight this methodological approach in their articles, making the contributions of these tools to biology less discoverable [3].

In this Essay, I reflect not only on tool access in low-resource settings, but also on building and developing appropriate open-source tools by local laboratories. To that end, international datasets are contrasted in Fig 1B to contextualize low-resource settings, not only in terms of funding, but also in their access to scientific and developer know-how, as well as industrial resources. Researcher density and the economic complexity index [4] were chosen as quantifiable variables to represent a country’s population-level “high-science” or “high-technology” environment, respectively (or both or none). They illustrate the know-how and recourse context a scientist in the country might face when purchasing and recruiting to equip a laboratory.

### Is it all about money?

If laboratories in low-resource settings have less funding and are more likely to use low-cost DIY technologies than laboratories in high-resource settings, then is the use of DIY technology simply a cost reduction strategy? The significant cost reductions that can be achieved with DIY and open-source hardware have been highlighted in the literature [5,6]. DIY hardware components often cost only 1% to 10% of commercial proprietary tools, offering substantial savings for low-resource laboratories and enabling new applications where the equipment cannot be simply reused; for example, building a microscope to be placed inside an incubator with pathogens. This cost reduction makes some open technologies popular budget tools. However, there are several other key aspects that influence the choice of using open technologies, particularly knowledge transfer advantages and local fabrication.

Knowledge transfer in a research context is broadly defined as the dissemination of knowledge, ensuring that research insights are used in practical terms. However, knowledge transfer is often narrowly interpreted as commercialization through patents and companies holding licenses. The recent growth of open hardware has led to an alternative model [7], in which open source designs allow for a faster and earlier adoption of new methods by scientific users [8]. Open-sourcing a design enables early adopters to build the new instrument before it might become commercially available, which, in turn, can lead to timely feedback and improvements to the original design. The motivational aspect for developers to speed up adaptation of their technology is important to mention alongside simple cost reduction of hardware and is likely to contribute to the growing popularity of open technology among developers.

### Local fabrication and availability are key

Another key aspect of technology choice is local production. In any research laboratory, access to 3D printers and a good connection to a CNC milling workshop can lead to faster instrumentation design turnaround times and a better ability to customize setups. For laboratories in low-resource settings, in particular, many suppliers and service providers are not available or easily accessible. Acquiring state-of-the-art equipment in such locations is more expensive and takes longer than it would in high-resource settings, often with uncertain shipping and import costs [9]. Furthermore, maintenance of such equipment is often unsustainable owing to the cost and availability of parts, a lack of specific know-how among local staff, and the expense of bringing the company’s maintenance team to the laboratory. These logistic and training challenges leave many instruments in low-resource settings out of order [10–12]. Therefore, the DIY approach can be the only affordable and, if well documented, sustainable option for laboratories in scientific environments characterized by economic and infrastructural constraints. In this context, the local control of versatile digital means of fabrication can make an important difference to the technology that can be made available in a laboratory. For example, it can allow researchers to 3D print the newest microscope stage model found online, or emailed from overseas, using standard printing filament supplies and about two days-worth of time [13]. Moreover, the world has recently witnessed the importance of local production as a result of the COVID-19 pandemic. In this moment of global shortage, many equipment-producing countries first supplied their own local needs, enforced by export bans or not being able to deliver elsewhere through collapsed trade routes. In response, many people spontaneously developed DIY and open-source solutions to locally produce personal protective equipment, such as 3D-printed face shields, and even medical instrumentation, especially mechanical ventilators [14,15].

Not only the low cost of tools, but also their availability in the limited portfolio of local sales and their reusability in different experiments are relevant. The global availability of the Arduino and Raspberry Pi boards, among others, is therefore also a likely contributor to their popularity for science in low-resource settings. DIY setups can also easily be automated and useful data export formats chosen without requiring specialized engineering knowledge. It must be noted that there are regions of the world where accessing a low-cost 3D printer and the laboratory members to operate it may be a challenge. These extreme resource challenges may be partially mitigated through frugal open technology, training, and core facilities, but an in-depth discussion of these topics goes beyond the scope of this article.

### The transformative potential of open hardware

Fig 1 suggests that DIY technology is widespread and likely used to access technology. But what exactly is the difference been “DIY technology” and “open technology”? Open-source hardware is defined as a physical object whose design is made publicly available so that anyone can study, modify, distribute, make, and sell the design or hardware based on that design [16]. In contrast to DIY, which is a practice, open hardware is a concept that describes how the design (which is inherently DIY in the eyes of the maker) is made available: It must at least have a documentation, modifiable design files, and a suitable license. More about best practices (see OSHWA) and standardization efforts [17] can be read elsewhere.

**Fig 2:**
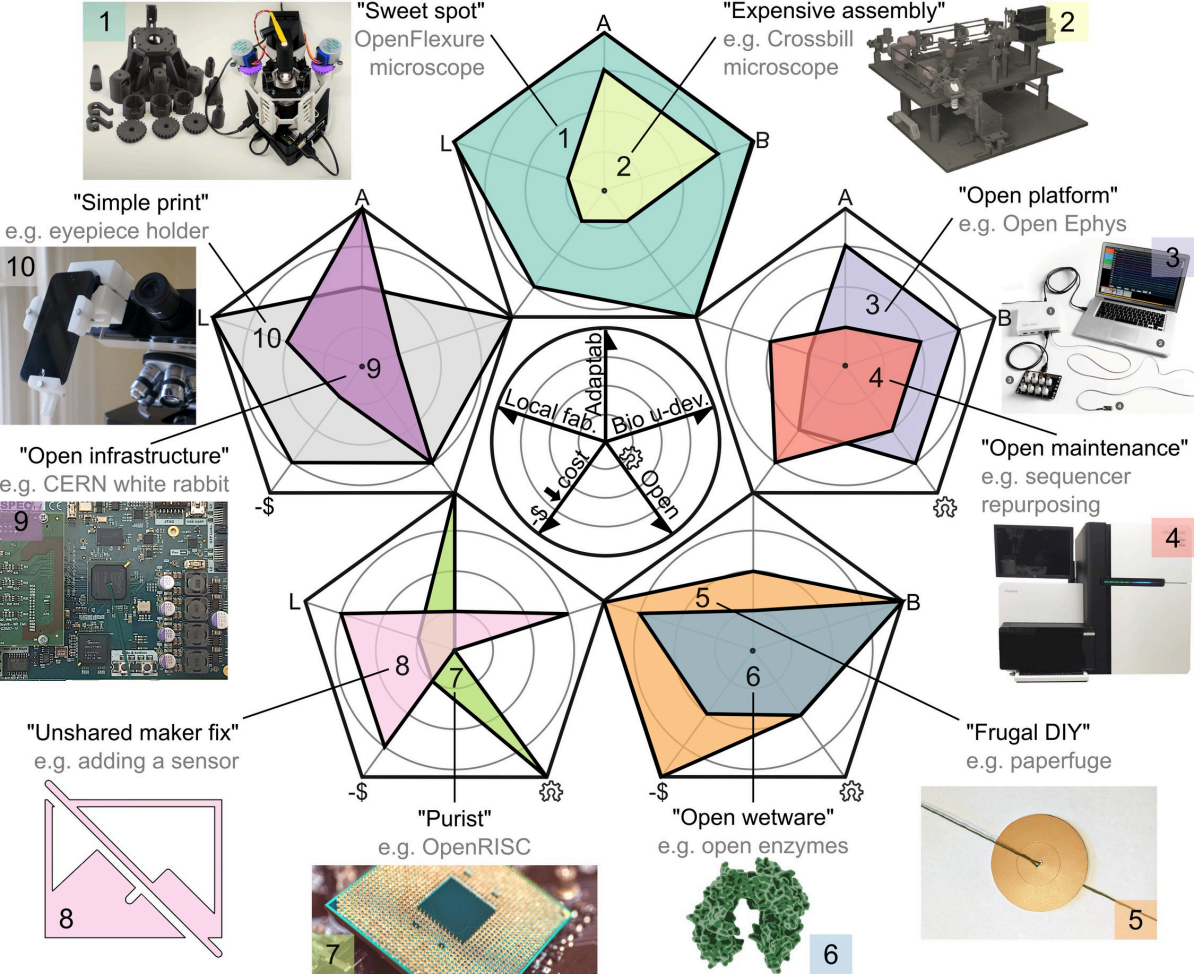
Ten types of open hardware. A range of projects falls within the portfolio of open hardware in a research context. Ten types (sweet spot, expensive assembly, open platform, open maintenance, frugal DIY, open wetware, purist, unshared maker fix, open infrastructure, and simple print) are conceptualized and rated in radar charts along five dimensions (see scale in the center): local fabrication suitability (can components be fabricated almost anywhere?); adaptability of the design; suitability for life science users to interact with the project documentation and contribute as developers; availability of modifiable open source design files; and to what degree needed components are low cost. The selected dimensions were rated subjectively on a scale from 0–4 to differentiate types of open hardware in general, not only the given examples [19–23]. See Box 2 for further explanation.

In practice, open technology can take a diverse range of forms and shapes (Fig 2). Box 2 categorizes open technology designs into 10 types to make sense of their differences in the context of this article. Even among researchers aiming to make their designs openly available, most end up not sharing design files for practical reasons [18], which makes these projects merely DIY, not open source. Open technologies span many “maker-style” life science projects, which can be particularly “make-able” when based on digital fabrication designs. Open technologies even include biological wetware, which also consists of physical objects and can even be digitally fabricated, e.g., in the case of enzyme encoding genes. Open-source technology can be low cost and simple, but can also be very technically challenging and expensive, such as the CERN particle accelerator electronics or computer processors.

**Box 2. Different types of open hardware.**

There are different types of open hardware for research, here categorized into the ten groups shown in Fig 2. Each has their own application advantages and challenges. In the “sweet spot” (1), e.g., the OpenFlexure microscope [19], hardware is suitable for local fabrication, is adaptable also by biologists, has digital design files, and is low cost, but few such designs exist to date. Many laboratories use the ease and low cost of the maker approach but never share solutions. “Unshared maker fixes” (8) is therefore a common category and is not actually open source. All other categories have the potential to meet the open-source hardware definition. This open nature even applies to instructions that may not contain any modifiable design files but nevertheless open up hardware know-how, e.g., “expensive assembly” (2), which consist of proprietary parts sold by science equipment providers. These components are usually expensive and cannot be self-fabricated, but through clever combinations, such equipment may give access to new methods and still save significant resources on comparable ready-to-use equipment [22]. Ready-to-use instruments may serve for modification or maintenance instructions in the “open maintenance” (4) category, which can only be made with a specific proprietary instrument [24]. Such projects may not require additional hardware components, and when they do, such components are often suitable for local fabrication. Science “open platforms” (3) such as Open Ephys [20] or OpenTrons [25] are modular and are usually moderately priced and commercially available instruments. On the downside, their instruments are usually optimized for industrial production and therefore do not lend themselves to local fabrication or part purchasing. “Frugal DIY” (5) projects are ultralow cost [26] and can be made from locally available materials; at the same time, they are usually limited in their applications and their lack of digital fabrication design limits their adaptability in research laboratories. They may also not comply with laboratory standard operating procedures, although they can open the door to science education for the general population. “Open enzymes” (6) and other bioreagents [27] are made available through an emerging effort to support the local production of such goods, but doing so is currently challenging and batch quality an issue for further local distribution. All aforementioned types are usually well suited for the involvement of biologists in their development, along with one more category, the “simple print” (10), which typically does not require much documentation or fabrication effort [28]. By contrast, “Purist” (7) projects aim to open up elementary hardware such as computer processors. Finally, open hardware does not have to be low cost and can involve “open infrastructure” (9), such as much of the electronic systems at CERN [29].

The open hardware concept is key to this discussion because it describes the difference between those internal designs in laboratories that simply take advantage of the ease of creating solutions “maker-style” and those that are shared in a way that is useful for others. Only open projects can be adapted to local needs and further developed to technology designs that lie beyond the development means of a single laboratory. The impressive growth potential of open-source projects has many examples in the older movement of free and open-source software, such as Linux and FIJI. Early examples of open technologies such as the OpenFlexure microscope [19] (Fig 2 and Box 2) provide us with a glimpse of where open hardware projects can go if they are built on digital design files and support the formation of a community. The OpenFlexure designs are open, low cost, high performance, and can be automated (e.g., for slide scanning). They are easy to adapt, and their designs use standard parts and modular digital files for local fabrication by 3D printing.

The mindset to use and build on each other’s knowledge to advance science is increasingly being applied to biology laboratory instrumentation. Although the documentation of individual projects may not always be perfect and community standards are still evolving [17], it is recommended that laboratories engage in this ongoing transformation, fail, learn, and try again with more success as a means of benefitting from the advantages for experimental research. Resources are available at all levels [3].

### Local development is about appropriate technology

It is necessary to discuss local fabrication and development not only in the context of its practicality, but also its meaning. Especially in the biosciences, new students might not be aware that building one’s own tools has multiple advantages and is becoming a common practice in laboratories worldwide. As already mentioned, this does not mean that the instrument has a low performance or is unreliable; rather, it is a useful strategy for technology control and innovation.

Most laboratories that self-identify as technology development teams are based in countries with many scientists and a complex industry (see countries in the top right-hand corner of Fig 1B) and have access to relatively large funding sources and industry collaborations. Such development projects are often undertaken with modular tools that are specifically commercialized for science developers and therefore look somehow “professional.” This includes black-anodized optomechanical parts, laser-modules with top-of-the-line branding, electronic automation boards running the supplier’s proprietary software libraries, the latest use-case-specific objective lens, and neat commercial sample holders. And why not? It can be an appropriate approach when funding allows, when there is stock at the local supplier and support teams offer customization. More importantly, these tools tend to be reliable and compatible and can thus save the team the time and hassle of making cheaper tools compatible, as well as double checking their performance. Replicating instruments from such assembly guides (type 2 in Fig 2 and Box 2) may or may not be cheaper than buying commercial alternatives, and they may or may not contain other open-source elements such as software or 3D design files. Still, useful instrumentation knowledge is disseminated, and, thanks to the modularity of tools, it can be further modified and improved.

By contrast, scientists who develop open technologies with a more ambitious definition of accessibility, with globally available tools and at lower cost, continue to face important symbolic and institutional restrictions, despite the multiple benefits for scientific and technological development of such an approach [30]. Such self-builds are sometimes viewed as unprofessional or unsuccessful in grant applications and, until recently, faced barriers from academic journals to publish their work. Fortunately, there are many good and well-published examples of open hardware that can be used to underline the scientific vision of such appropriate technology [31–39]. Still, this stigma has implications for sharing, as the developers might not feel that their solutions are proficient enough to share, and they might not self-identify as technology developers. The term “appropriate technology” is useful to explain why many such solutions are nevertheless of interest to scientists in high-resource and low-resource settings alike. A technology is deemed to be appropriate when it is “compatible with local, cultural, and economic conditions (i.e., the human, material, and cultural resources of the economy) and utilizes locally available materials and energy resources, with tools and processes maintained and operationally controlled by the local population” [40] and, when possible, open source [41]. Tools that may be appropriate in a few well-resourced countries may be difficult to obtain, maintain, and afford in other countries, making technologies based on these tools inappropriate for research use elsewhere. Technology development labs that have access to a wide array of tools may use lower-cost technology to enable new applications, or consider global access to components in order to improve opportunities for global adoption of their appropriate technology and thereby increase the impact of their methods. Either way, such technologies may simply be “appropriate” for the task. For reasons of cost, access, and operation, it may not be appropriate to, for example, use professional chemically resistant microfilters to fish microplastics from the ocean off the shores of Newfoundland, and instead, baby’s tights may be the right tool for the job [42]. Digital fabrication is also an appropriate approach to build technology for research in a wide range of locations globally. It is therefore recommended to assess whether a tool is an appropriate technology, given its intended application context, rather than just highlighting its low cost or even using the negatively connotated term “cheap.” If highlighted as appropriate technology, designs in manuscripts are less likely to miss their audience.

### Open technology trends in biology laboratories

**Fig 3:**
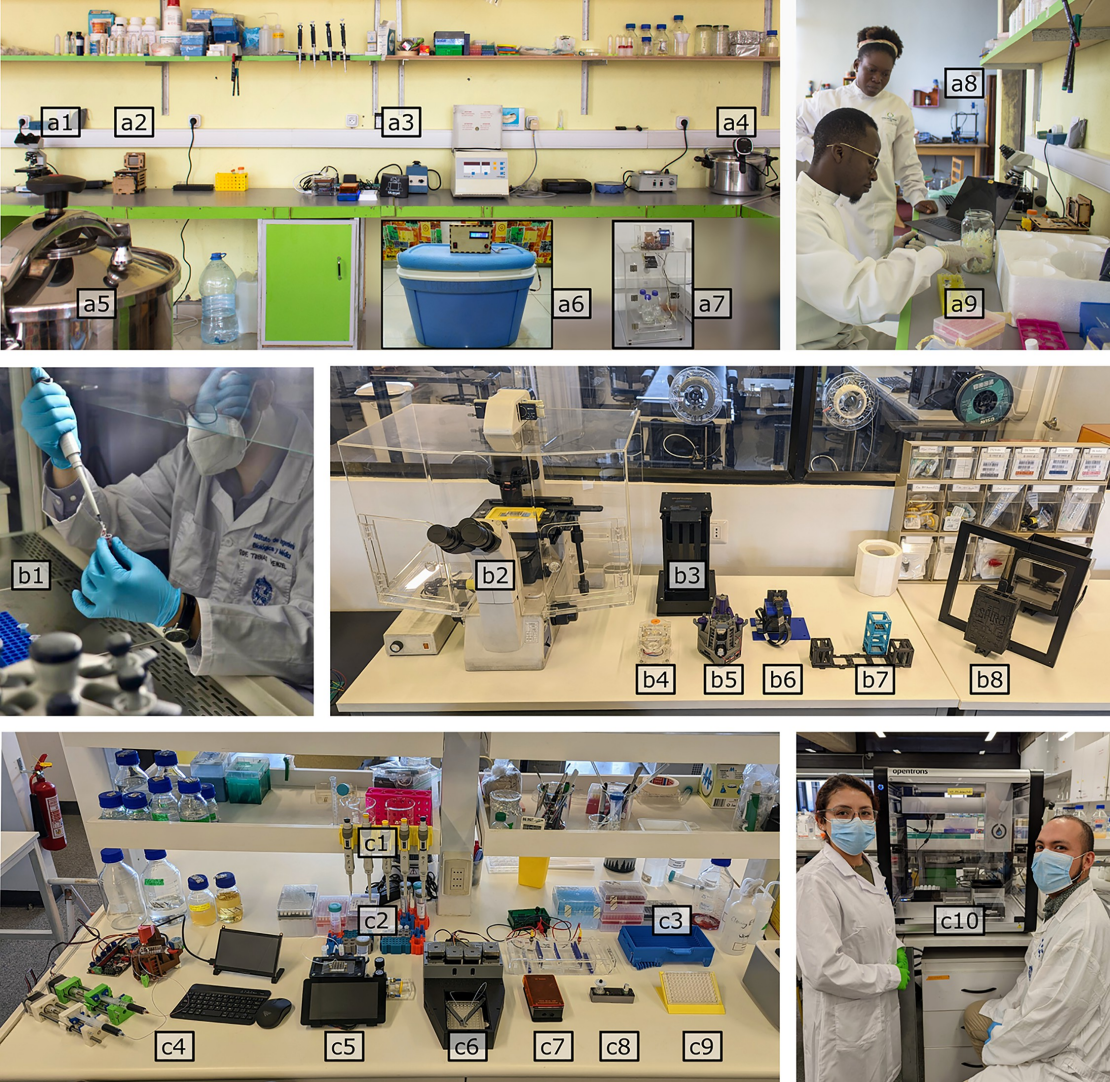
Examples of biology laboratories equipped with open technology. The top images show the Mboa Lab in Cameroon (source: openbioeconomy.org) and middle and lower images show the Wenzel Lab in Chile. The following equipment is shown: basic commercial light microscope (a1), open hardware thermocycler (rebuild of OpenPCR) (a2), small commercial DNA analysis equipment (miniPCR, bluegel, vortexter, centrifuge) (a3), repurposed Sous Vide as water bath (a4), repurposed pressure cooker as autoclave (a5), self-designed open hardware incubator (a6), self-designed open hardware shaking incubator (a7), commercial open hardware 3D printer (a8), use of reagents and open enzymes (a9). Secondhand commercial laminar flow hood (b1), secondhand commercial microscope with custom enclosure and 3D-printed sample and camera adaptors (b2), open source laser-cut fluorescence plate imaging station (rebuilt; [48]) (b3), open laser-cut smartphone microscope (Roachscope from Backyard Brains) (b4), open 3D-printed OpenFlexure microscope in fluorescence configuration (rebuilt and star-LED fitted; [49]) (b5), 3D-printed computational microscope (rebuilt with Unicorn Raspberry Pi hat) (b6), open 3D-printed holographic microscope (UC2 rebuild; [31]) (b7), open time-lapse multiple Petri plate imaging system (SPIRO rebuild in progress; [50]) (b8). Student-designed 3D-printed pipette holders with earthquake safety (c1), open 3D-printed tube racks (rebuilt from Dormant Biology Lab design) (c2), 3D-printed pipette tip aligner for tip box refill (Elster rebuild) (c3), self-designed microfluidics educational workstation (Raspberry Pi–based control of 3D-printed syringe pumps and microscope) (c4), open workstation for integrated single-cell transcriptomics (RNA-seq miniDrops rebuild; [33]) (c5), open 3D-printed microscopy pipetting robot that can be mounted on a microscope (rebuild in progress; [32]) (c6), open-source gel electrophoresis set (self-designed parametric laser-cut gel chamber with open commercial IORodeo power supply and IORodeo blue LED transilluminator) (c7), open thermocycler (Gaudilab PocketPCR built from commercial set) (c8), open self-designed well-plate locator stand (c9), open commercial pipetting robot OpenTrons (c10).

In recent years, a broad portfolio of open technology designs has been made available, enough to equip entire life science laboratories (Fig 3). In this Essay, general-purpose equipment is highlighted for broad interest and as a good way to start engaging in open technologies. In addition to examples in Fig 3, many other examples have been published, including a 3D-printable rotator mixer for incubators [43], a portable CO_2_ incubator for tissue culture [44], a 3D-printable spectrophotometer [45], an anaerobic chamber with a DIY catalyst [46], a system for automated parallel microbial cultivation [36], an isothermal well-plate reader for LAMP reactions (MIRIAM), an open source Prusa 3D printer modified for bioprinting [47], and many more. Besides integrated and automated equipment, there is a wealth of simpler open-source 3D-printable designs, such as equipment adapters, covers, clips, sample or pipette holders, well-plate locator stands, physiological models, and more (see Thingiverse, Instructables, Hackster.io, Wikifactory, and other platforms). These can be redesigned or adapted to local needs. For example, this semester some of my students designed 3D-printed pipette holders that hold the pipettes in place with a gentle flex mechanism (Fig 3C1), so that they are less likely to fall during Chile’s frequent shakes.

General-purpose equipment designs tend to be of broad interest, but often commercial and proprietary alternatives already exist. In contrast, open solutions particularly stand out for solving special applications that few other people currently share, or where equipment cannot be recovered, e.g., outdoor sensors and technology for infectious disease research [49,51]. Another appropriate approach for biological experiments is to use low-cost electronic boards to automate or add sensors to experiments, e.g., for humidity, pressure, temperature, or positioning. In this context, it often does not matter to the researcher if the board itself is open source (e.g., Arduino) or not (e.g., Raspberry Pi), it is simply used as a component in an otherwise hopefully well-documented setup. Examples and approaches for open electronics in biology have recently been described elsewhere [3]. Two particular active areas of open development are microscopy [52] and neuroscience. Another exciting area of growth is open wetware, which democratizes access to enzymes, reagents, and reagent fabrication designs [27,53,54]. Automation for sample handling and calibration is furthermore used to improve reproducibility of research.

Currently, it is rare to see a laboratory with many open technologies used at the same time. Usually, even in low-resource settings, laboratories contain a mix of some relatively new instruments funded by research grants, secondhand equipment (usually older, borrowed from colleagues, moved from other laboratories or projects, and some donated), and self-made solutions, ranging from buffer-mixes, coat hangers, and incubator racks, to the more complex research instrumentation discussed above. The mix of technology types in use is also illustrated in the laboratory examples from Africa and South America in Fig 3. Importantly, expensive setups can still be an option in low-resource settings, although this usually occurs in the context of core facilities when they are made available to other research users that pay for use or as a local service.

### From the laboratory to industry

Engaging in open technology development is not meant to oppose the mainstream approach of knowledge transfer through commercialization. As with multibillion dollar open software companies, nonproprietary products can still be sold, marketed, developed, and serviced. All these are valuable ways of making an open design even more available. Open hardware–related business models are an ongoing area of development [55,56] and practice, as demonstrated by the examples of open hardware companies provided in Table 1.

**Table 1:** Examples of open hardware companies relevant for biology laboratories.

Company name	Field or product	Link
Tindie	Online marketplace for open electronics and hardware products. Sales platform for makers and small businesses	https://www.tindie.com/
SparkFun	Designs, manufactures, and sells open microcontroller development boards and breakout boards	https://www.sparkfun.com/
Seeed Studio	Integrated platform for creating hardware solutions for IoT, AI, and Edge	https://www.seeedstudio.com/
Sci-Bots	Dropbot, digital microfluidic control system	https://sci-bots.com/
Sanworks	Automated systems for neuroscience research	https://sanworks.io/
Safecast	Crowdsourced environmental data with open hardware, originally radiation in Japan now also air quality globally	https://safecast.org/
Red Pitaya	FPGA-powered boards as multifunction laboratory and engineering instrument	https://redpitaya.com/
Public Lab	Community science tools for environmental monitoring	https://www.publiclab.org/
Prometheus Science	Open hardware for biology consulting	https://prometheus-science.com/
Precious Plastic	Plastic recycling equipment	https://preciousplastic.com/
OpenTrons	Pipetting robot and addons	https://opentrons.com/
OpenQCM	Quartz Crystal Microbalance, e.g., for protein affinity measurements at molecular resolution	https://openqcm.com/
OpenFlexure Industries	Manufactures the openflexure microscope and openflexure block stage	https://openflexure.com/
Open Ephys	Electrophysiology laboratory tools	https://open-ephys.org/
OpenBCI	Brain–computer interface platform	https://openbci.com/
Open Acoustic Devices	Designs supports and deploys acoustic hardware and software for environmental and wildlife monitoring projects, audio moth	https://www.openacousticdevices.info/
Olimex	Developer and provider for development tools for embedded market, including non-open boards	https://www.olimex.com/
neurogig	Instruments for neurophysiology laboratories	https://neurogig.com/
Kitspace	Share and order electronic projects with the automatic component shopping list generator for github projects	https://kitspace.org/
IORodeo	Designing and selling biolab hardware, now mainly potentiostats	https://iorodeo.com/
Elphel	Imaging hardware and software	https://www.elphel.com/
Beneficial Bio	Network of social enterprises for biolabs	https://beneficial.bio/
BeagleBoard	BeagleBone computers	https://beagleboard.org/
Backyard Brains	Demonstration instruments for neuroscience education	https://backyardbrains.com/
Arduino	Designs and manufactures single-board microcontrollers and microcontroller kits for building digital devices	https://www.arduino.cc/
Apertus	AXIOM cameras	https://www.apertus.org/axiom
Adafruit	Designs, manufactures, and sells a number of electronics products, electronics components, tools, and accessories	https://www.adafruit.com/
3D printer providers such as Prusa (Prusa Research), LulzBot (FAME 3D), Voron, Rostock Max (seemecnc)	Providers of open source commercial 3D printers	https://www.prusa3d.com https://lulzbot.com https://vorondesign.com https://www.seemecnc.com

Often academics and universities do not have the means, legal or industrial knowledge to enforce global intellectual property infringements. Publishing designs openly can protect from future patent claims by others. And while patents are still a good marketing tool for investors, the several year cycle of patent processing is often out of sync with fast-moving technological development. The investing markets might also not be the best place to solve local problems; different business models could be used without the pressure of venture capital to make a difference closer to home. Open projects can be faster, more flexible, and, perhaps most importantly, closer to the users. Users can directly modify and improve the open technology at hand, thus advancing technology, contributing to local availability of solutions, and empowering citizens [57–59].

### Barriers to adopting open hardware

As discussed above, biology laboratories in low-resource settings face challenges around technology access that are far more complex than the availability of money to buy instruments. Open-source designs and digital local production are increasingly employed as promising solutions to several of these challenges. So why is open technology not yet actively supported by funders and institutions in these regions? There is no conclusive data available to answer this question rigorously. The following points might contribute to understand the status quo: Open hardware sharing is a relatively recent concept and not yet known by all; scientific development in low-resource settings tends to follow the patterns of scientific development in high-resource settings, where standards of open hardware have rarely been implemented at an institutional level but first examples are emerging [60]; and low-resource settings tend to have a lower density of scientists and a less complex economy and, therefore, less complex local production facilities than high-resource settings (Fig 1B, bottom-left). This lack of a technology development ecosystem results in reduced access to production resources and qualified personnel to build instruments. Fabrication skills are highly sought after and knowledgeable individuals can contribute to the migration of advanced human capital away from low-resource settings. This insight into the local ecosystem highlights the need for detailed documentation and the use of standardized and accessible tools to enhance regional application.

Many individuals and laboratories in low-resource settings do not see themselves as technology developers and, therefore, perhaps do not consider their local solutions as worth sharing. In fact, in many places, foreign and western brands are highly valued, which can extend to laboratories and their heads wanting to be seen as professionals by working with popular international equipment. Furthermore, in China [61,62] and some other parts of Asia, complex production facilities and technical know-how are available and enable fast and low-cost prototyping of technology, but designs and calibration instructions are often not shared in detail. The lack of sharing of documentation may be caused by a belief that people anywhere could easily build something equivalent, or by a different mindset around intellectual property licensing, technology transfer, task-sharing, communication, and documentation practices.

We could see an even faster growth of custom life science technology designs if we adjusted institutional policies [60] to incentivize departmental workshops to release the custom designs [18] they create for individual researchers. Often, the opposite is the case, and institutional workshop staff are not allowed to release files or allow the respective academic to make the files available. Sometimes, academics are also prevented from open-sourcing their potentially patentable developments. This is one more reason to work openly from the start of a project and release documentation as the research develops, enabling early collaboration and feedback. With such an approach, there is no point in time at which an institutionally problematic amount of intellectual property is released. The “open from the start” model also avoids the backlog of documentation tasks left for final deadlines.

## Conclusions

Open technology can be a powerful strategy to access appropriate research technology in both low-resource and high-resource biology laboratories. This fast-growing strategy generally does not aim to break with the tradition of proprietary and commercial approaches; instead, it adds a new dimension to current mainstream knowledge transfer models. Supporting openness can lead to a transformative increase in technology access and local development, especially in settings that are currently challenged in this regard. This new, fast, user-centered, open approach promises a place at the global research table for low-resource laboratories, and more widespread access to appropriate research technologies. It enables the local development, adaptation, and usage of technologies that are appropriate for local contexts and enhance regional innovation and business. A large portfolio of designs already exists with a broad spectrum of design approaches, cost, commercial availability, and complexity, with something to offer for anyone who would like to become an open technology user and developer. If we limit ourselves to the current mainstream practice of low-resource laboratories to mainly buy or inherit old, basic, or hardly maintainable equipment, regional bioscience researchers will most likely struggle and miss the opportunity of being at the forefront of scientific development in the future. Every open hardware user and builder means a local gain of good examples, technological know-know, and infrastructure enablers, in a way that users of proprietary, closed technology can never be. Future changes in relevant institutional guidelines and policies from funding bodies worldwide should aim to recognize, encourage, and enable an open technology approach. Such institutional recognition and the availability even of relatively small flexible funds can make a large difference to laboratories assembling open technology.
